# Prevalence of *Chlamydia trachomatis* Infection and Its Association with Sexual Behaviour and Alcohol Use in the Population Living in Separated and Segregated Roma Settlements in Eastern Slovakia

**DOI:** 10.3390/ijerph14121579

**Published:** 2017-12-14

**Authors:** Ingrid Babinská, Monika Halánová, Zuzana Kalinová, Lenka Čechová, Lýdia Čisláková, Andrea Madarasová Gecková

**Affiliations:** 1 Department of Epidemiology, Faculty of Medicine, Pavol Jozef Šafárik University in Košice, 041 80 Košice, Slovakia; ingrid.babinska@upjs.sk (I.B.); zuzana.kalinova@upjs.sk (Z.K.); lenka.cechova@upjs.sk (L.Č.); lydia.cislakova@upjs.sk (Lý.Č.); 2 Department of Health Psychology, Faculty of Medicine, Pavol Jozef Šafárik University in Košice, 040 11 Košice, Slovakia; andrea.geckova@upjs.sk

**Keywords:** Roma, ethnicity, *Chlamydia trachomatis*, sexual behaviour, alcohol use

## Abstract

The aim of the study was to explore sexual behaviour and the occurrence of *Chlamydia trachomatis* (CT) infection in the population living in Roma settlements compared to the majority population in Slovakia and to assess the association between alcohol use and sexual behaviour within both populations. A cross-sectional population-based Hepa-Meta study was conducted in Slovakia in 2011. The final sample comprised 452 Roma and 403 non-Roma respondents. The occurrence of CT was detected by direct proof of the pathogen by PCR. The association between alcohol use and the prevalence of risky sexual behaviour were assessed using a logistic regression. First intercourse at age 15 or younger was reported by 27.9% of Roma (vs. 4.5% of non-Roma); 93.4% of Roma (vs. 77.9% of non-Roma) used condom inconsistently, 22.8% of Roma (vs. 43.9% of non-Roma) used a condom for protection from unwanted pregnancies and only 8.8% of Roma (vs. 21.8% of non-Roma) due to protection against infectious diseases. However, Roma reported having had five or more sexual partners less often compared to the majority (11.5% of Roma vs. 20.6% of non-Roma). Binge drinking at least once a month was associated with a higher number of sexual partners in both groups, but not with condom non-use. The prevalence of CT infection in the Roma population was higher (3.8%) compared to non-Roma (2.7%); however, the difference was not statistically significant. Our study found no differences in the prevalence of CT infection between Roma and non-Roma despite differences in sexual behaviour. Roma begin their sexual life earlier and have unprotected sex more often, but on the other hand, they seem to be much more restrained in terms of the number of sexual partners compared to the majority population.

## 1. Introduction

The Roma population ranks among the largest ethnic minority groups living in Slovakia. According to census data, the number of people who declared themselves as Roma increased from 1.4% (75,802 citizens) in 1991 to 2% (105,738 citizens) in 2011. This, however, is only about a quarter of the estimated Roma population in Slovakia, which ranges between 8 to 10% (around 320,000–500,000 Roma citizens) of the country’s total population [1,2]. The territorial distribution of the Roma population in Slovakia is uneven, with eastern Slovakia being among the areas with the highest number of Roma [1,2].

The Roma population living in separated and segregated settlements can be considered as the most disadvantaged group, regarding the poor socioeconomic position, living and working conditions, segregation and social exclusion [3,4,5,6,7,8]. Several studies have revealed a range of barriers for Roma in accessing health services [9,10,11], including sexual and reproductive health services [12]. These conditions have serious consequences for their health outcomes in terms of less healthy lifestyles [13], poorer self-rated health [9], higher morbidity from both infectious and chronic diseases and shorter life expectancy [3]. The different character of sexual and reproductive behaviour of this population, e.g., the early initiation of sexual life and reproduction and unprotected sexual contact, could result in a different incidence of sexually transmitted diseases.

*Chlamydia trachomatis* (CT) infection is the most commonly reported bacterial sexually transmitted disease (STD) in the world [14]. In women, it is the cause of mucopurulent cervicitis, salpingitis, endometritis and urethritis. In men, the most common illness resulting from CT infection is urethritis, and complications include epididymitis, prostatitis and proctitis. However, most CT infections have minimal symptoms or are asymptomatic, including extragenital infections of the oropharynx and rectum [15]. Repeated and untreated CT infections can lead to serious complications, such as chronic pelvic pain, infertility and ectopic pregnancy. In addition, during pregnancy they might lead to newborn diseases, such as infant conjunctivitis and pneumonia [16,17]. Several studies have confirmed that chlamydial infection of the genital tract facilitates the transmission of human immunodeficiency virus (HIV) [18,19]. CT is also one of the common causes of reactive arthritis [20,21].

CT infections are reported more often in women than in men. Two-thirds of all CT infections are reported in young people between 15 and 24 years of age [22,23]. Other factors associated with chlamydial infection include sexual risk behaviour, such as multiple lifetime sexual partners, sex with symptomatic partners, inconsistent condom use and history of previous or coexisting sexually transmitted diseases (STD) [22,24,25]. Moreover, inequities in social and economic conditions are additional risk factors, which are reflected in the differences in the prevalence of STDs [26,27,28,29]. Surveillance data show higher rates of reported STDs among some racial or ethnic minority groups in comparison with the majority population [26,28,30,31].

The association of sexually transmitted infections with risky sexual behaviour is obvious, but particular populations (ethnical, cultural, social) differ in onset as well as in prevalence of particular sexual behaviour (e.g., early initiation of sexual life, number of sexual partners, condom use). Moreover, risky behaviours tend to cluser or moderate each other, e.g., binge drinking might stimulate other risky behaviour (unsafe driving or unsafe sexual behaviour) which may result in adverse health outcomes (injuries, sexually transmitted infections). Binge drinking is indeed associated with risky sexual behaviours (anal sex, multiple sex partners), sexually transmitted infections and unintended pregnancy [32,33,34]. Some cross-sectional [35,36,37] and longitudinal studies [38] indicate that young people who tend to have multiple sexual partners over time are at increased risk of unprotected sex and they have more permissive values on sex and an elevated proportion of homosexual behaviour [38].

In Slovakia, surveillance data on the prevalence of CT infections are based on cases reported to Regional Public Health Authorities. Almost 900 CT cases are reported annually. The five-year average (2012–2016) amounted to 867.8 CT infections per year [39]. It is assumed that the occurrence is considerably higher, as due to the asymptomatic nature of the infection, many of them are not diagnosed or reported. In addition, these reports do not include racial/ethnic information of the patient. Therefore, there are only limited data on the occurrence of CT infections in ethnic groups, which indicates a need for further research. 

The aim of our study was to explore sexual behaviour and occurrence of CT infection in the population living in separated and segregated Roma settlements in comparison with the majority population in Slovakia. Moreover, the study aimed to assess the association between alcohol use (binge drinking) and sexual behaviour within the Roma and non-Roma populations.

## 2. Materials and Methods

### 2.1. Study Population

**Figure 1:**
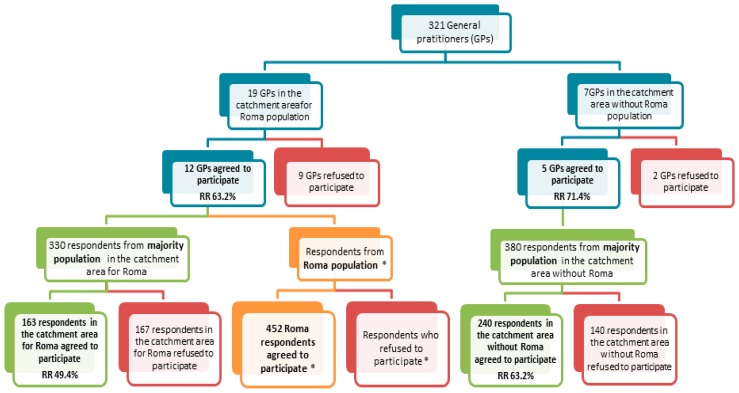
Recruitment of the Roma and non-Roma population (GPs: general practitioners; RR: response rate; * missing data on response/non-response in Roma).

We used data from the cross-sectional Hepa-Meta study conducted in the eastern part of Slovakia in 2011. The aim of the study was to map the prevalence of viral hepatitis B/C and metabolic syndrome in the population living in separated and segregated Roma settlements and to compare it with the occurrence of the same health indicators in the majority population, considering selected risk and protective factors of these health indicators [40]. This cross-sectional population-based study was set up following the principles of community-based participatory research. The target population comprised residents of settlements in the Košice region aged 18–55 years, and the control group was the majority population in the same region and of the same age composition. From 30 general practitioners (GPs) who provided primary care for the inhabitants of these settlements we randomly selected 19 who were contacted, and 12 agreed to participate (response rate 63%). For the majority population in the catchment area, we randomly chose seven general practitioners from a list of general practitioners in the selected area without a nearby Roma settlement. Five agreed to participate in our study (response rate 71%). Roma were recruited via local Roma community workers. From all Roma who were present in the settlements and received information about our study, 452 chose to participate. Since the recruitment of Roma respondents was carried out under the unpredictable conditions in Roma settlements, we were not able to compute the response rate. People from the majority population were randomly chosen from the GP lists of patients. They were contacted via phone and mail by trained research assistants, who provided information about our study and invited them to participate. A total of 403 agreed to participate in our study (response rate 56.8%). Recruitment of the Roma and non-Roma population is visualised in Figure 1.

Trained medical personnel collected the blood and urine samples and performed anthropometric measurements in the ambulance of the cooperating GPs. For the majority population, trained assistants were present in the ambulance to assist with questionnaires if needed. In Roma respondents, questionnaires were administered in community centers by community workers or trained assistants who provided help in case of limited literacy, which seem to have the smallest impact on validity of the data [41].

The study was approved by the Ethics Committee of the Faculty of Medicine at Šafárik University, Košice (No. 104/2011). Participation in the study was on a fully voluntary basis and anonymous. Detailed information about the study and its procedures was given to all respondents, and informed consent was obtained prior to the medical examination.

The sample covered 452 residents aged 18–55 from Roma settlements in the Kosice Region in Slovakia. The control group included 403 residents from the majority population in the same region and in the same age range. A total of 422 Roma and 335 non-Roma respondents were screened for the presence of the CT bacteria.

### 2.2. Questionnaire

The questionnaire gathered data about participants’ sex, age, school grade, sexual behavior and alcohol use.

Highest education was measured by asking respondents the question “What is your highest educational degree attained?” Possible responses were unfinished or finished elementary/apprenticeship/secondary/university. We merged the last two categories into one category: higher education. Respondents were asked if they are currently employed (except for community service) or unemployed.

Respondents were asked whether they currently live with a partner (husband, life partner) or not (single, divorced, widow), if they had ever had sexual intercourse (yes/no), at what age they had their first sexual intercourse (15 years or less/16 years and over), how many sexual partners they have had so far (1/2–4/5–9/10–24/25 and over), how often in the last year did they use condoms during sexual intercourse (always/almost always/rarely/never), what was their reason for condom use (prevention of unwanted pregnancies/protection against communicable diseases) and if they have ever had sexual intercourse for money or some other reward (yes/no). The answers to the question how often in the last year did they use condoms during sexual intercourse were dichotomised to consistent (always) vs. inconsistent use (almost always/rarely/never).

Alcohol consumption was measured by asking about binge drinking—the consumption of 6 or more doses of alcohol on one occasion (1 dose = 0.5 L of beer, 0.2 L of wine or 0.05 L of spirits). Possible responses (never/less than once a month/once a month/once a week/daily or almost daily) were dichotomised in two categories: less than once a month/once a month or more [42,43].

### 2.3. Urine Samples Measurements

The occurrence of CT was detected by direct proof of the pathogen in the first portion of urine samples by polymerase chain reaction (PCR) using the commercial DNA-sorb-AM nucleic acid extraction kit and the AmpliSens^®^ *Chlamydia trachomatis*-EPh PCR kit (the Federal Budget Institution of Science, Moscow, Russia). Extraction and purification of DNA as well as PCR analysis were performed according to protocols recommended by the manufacturer. A sample was considered to be positive for CT DNA if the 330-bp band was present in the gel.

### 2.4. Statistical Analysis

We used basic descriptive statistics for analysis of the obtained results. The *t*-test was used to assess differences in the mean values and the Chi-square test to assess differences in proportions. Next, we examined the association between Roma ethnicity and sexual behaviour and alcohol use, and the association between alcohol use (binge drinking) and sexual behaviour using logistic regression models adjusted for age (enter method). The dependent variables were living with a partner in the present, age at first sexual intercourse, number of sexual partners, condom use, reasons for condom use, sexual intercourse for money or reward and drinking behaviour. The independent variables were Roma/non-Roma status and binge drinking (less than once a month/once a month or more). The analyses were performed with SPSS Statistics version 20 (IBM, Armonk, NY, USA).

## 3. Results

### 3.1. Basic Characteristics of Sample

As shown in Table 1, the sample consisted of 452 Roma (mean age = 34.7, standard deviation (SD) = 9.14, 35.2% men) and 403 non-Roma (mean age = 33.5, SD = 7.4, 45.9% men). Respondents living in Roma settlements in comparison with non-Roma respondents reported significantly more frequently a lower level of educational attainment and being unemployed.

### 3.2. Sexual and Drinking Behaviour

Upon examination of the sexual behaviour of the population living in separated and segregated Roma settlements (Table 2) we found that 82.7% of Roma reported currently living with a partner, 27.9% had their first intercourse at age 15 or younger and 11.5% indicated that up to now they had had five or more sexual partners. Over 93% of Roma reported unprotected intercourse without using a condom, with 22.8% of Roma using one for protection against unwanted pregnancies and 8.8% of Roma in order to protect against infectious diseases. Another 2.9% of Roma participants reported that they have previously had sex for money or some other reward.

In comparison to the majority population, Roma had 2.5-times higher odds of living with a partner and 7.7-times higher odds of having their first intercourse at age 15 or younger. Significant differences by ethnicity were also found regarding condom use. Roma had 3.5-times higher odds of inconsistent using a condom, and almost 3-times lower odds of condom use to protect against unwanted pregnancies or protect against infectious diseases. However, less sexual risk behaviour was recorded among Roma in comparison to the majority population with regards to the number of previous sexual partners. Roma had more than two-times lower odds of having five or more sexual partners.

We found no significant differences in alcohol consumption between Roma and non-Roma regarding the frequency of binge drinking of six or more doses of alcohol on one occasion, with 16.6% of Roma (15.6% of non-Roma) reporting drinking six or more doses of alcohol on one occasion once a month or more often.

Our analysis of the relationship between binge drinking and sexual behaviour (Table 3) in the population living in Roma settlements, proved that Roma who reported a frequency of binge drinking at least once a month had seven-times higher odds of having five or more sexual partners compared to Roma with a lower frequency of binge drinking (odds ratio (OR): 6.99; 95% confidence interval (CI): 3.73–13.09). In the Roma population condom use was not significantly affected by the frequency of binge drinking. Similarly, non-Roma respondents who reported a frequency of binge drinking at least once a month had higher odds of having five or more sexual partners compared to respondents who reported a lower frequency of binge drinking (OR: 4.32; 95% CI: 2.35–7.78). In the non-Roma population, binge drinking at least once a month was not associated with condom use.

### 3.3. Occurence of Chlamydia Trachomatis Infection

A total of 757 respondents were screened for CT infection. CT positivity was found in 25 (3.3%) participants, 16 (3.8%) from the Roma population and 9 (2.7%) from the majority population. The highest positivity was detected in Roma women (4.8%). However, we did not find a significant difference between positive cases in the Roma and non-Roma populations (Table 4). Exploring the association between sexual behaviour and the occurrence of CT infection was not possible due to the low number of CT-positive respondents.

## 4. Discussion

The purpose of this study was to explore the differences between Roma and non-Roma regarding sexual behaviour, alcohol use and the prevalence of CT infection. Furthermore, we aimed to assess the association between binge drinking and sexual behaviour. 

Consistent with other studies, we found more unfavourable socioeconomic characteristics among Roma compared with non-Roma regarding education and unemployment [3,5,8].

The investigation of differences in sexual behaviour between Roma who live in settlements and the majority population from the same region in Slovakia confirmed the higher incidence of first intercourse at age 15 or younger among Roma. The young age of initiation into sexual life in the Roma minority is almost always followed by development of pregnancy and childbirth at an early age [44]. Previous studies have pointed out the early start of sexual life among Roma as well as the fact that Roma women have a higher total fertility rate and adolescent birth rate when compared with non-Roma women [44,45,46,47]. In Slovakia, the fertility of Roma women in the years 1992–2012 in all age groups was significantly higher than that of non-Roma women. The biggest difference was found in the younger age, where the level of fertility for women to 22 years old in Roma localities was more than three-times higher in comparison with non-Roma women [46]. Several ethnographic and sociological studies have pointed to the fact that having many children has long been the most important traditional life value for Roma, and the success of a marriage is assessed according to the number of children born [46]. On the other hand, the results of censuses in Slovakia (1980, 1991, 2001 and 2011) confirm that the younger generation of Roma who publicly demonstrate belonging to this ethnic group are more inclined to two and especially three children, and the number of families with a high number of members is reduced [46]. In our study the average number of children in a household was significantly higher in Roma families (3.8 children) than in non-Roma (1.2 child).

Our research found very high rates of unprotected sex among the Roma population. Nine out of ten Roma reported inconsistent using a condom. This is consistent with previous studies, which found high rates of unprotected intercourse among Roma in Slovakia [48], Bulgaria [49,50,51,52] and Hungary [52] and among Roma sex workers in Serbia [53]. In this study, protection against unwanted pregnancy was a more common reason for using a condom than protection against infectious diseases. This is probably related to the lack of knowledge regarding STDs, transmission and preventive measures in the Roma population [47,52], though qualitative research provides another possible explanation. Marston and King (2006) identified in their systematic review seven key themes that may help in understanding young people’s sexual behaviour in regard to unsafe sex. They are: young people assess a potential sexual partner as “clean” or “unclean”; sexual partners have an important influence on behaviour in general; condoms are stigmatising and associated with lack of trust; gender stereotypes are crucial in determining social expectations and, in turn, behaviour; there are penalties and rewards for sex from society; reputations and social displays of sexual activity or inactivity are important; and social expectations hamper communication about sex [54].

Our finding that a higher frequency of binge drinking (once a month or more) is associated with a higher number of sexual partners (five or more partners) is in line with previous research. Alcohol and substance use may reduce inhibitions and impair judgement in these users, which can manifest on their sexual behaviour regarding unplanned sex, having first sex with their last partner after they had only recently met or multiple partners, sexual activity in early teenage years and also on their poor sexual health outcomes [55,56,57,58,59], including chlamydial infection [60].

However, the evidence of a relationship between alcohol use and condom non-use are inconsistent. Some studies observed a positive association between drinking and a greater likelihood of unprotected sex [49,61,62]. On the other hand, several studies found no evidence of a relationship between alcohol use and condom non-use and suggested that alcohol use may not be directly responsible for condom non-use [63,64]. We have not confirmed an association between higher frequency of binge drinking and condom non-use. This association was not found in Roma. 

Several studies point to ethnic differences in the prevalence of STDs [27,28,31]. In the United States, non-Hispanic blacks had a 1.5-times higher risk for chlamydia and five-times higher risk for gonorrhea than non-Hispanic whites [28]. In Great Britain, a significant association was recorded between ethnic origin and reported STDs in the past five years, with increased risk in the sexually active black Caribbean and black African (only men) populations compared with the white population [31]. Similarly, a higher CT prevalence was found among Surinamese/Antilleans in comparison with participants with a Dutch background in the Netherlands [27]. Surprisingly, the results of a community-based survey in Serbia found a low prevalence of STDs (HIV infection, hepatitis C and syphilis) among Roma youth, despite the high prevalence of reported risky behaviours [47]. In our study the prevalence of CT infection was 3.8% in the population living in Roma settlements and 2.7% in the majority population; however, ethnic differences were not significant. This finding is in line with previous studies in Slovakia, which reported the prevalence of this infection in the Roma population between 3.9 to 4.4% [65,66]. A slightly higher prevalence (5.2%) was found among men in Roma sociocentric networks in Bulgaria [49]. Matser (2013) and Fenton (2005) point out that sexual behaviour alone cannot explain the differences in STD prevalence among ethnic groups [27,31]. The higher CT prevalence among Surinamese/Antilleans in comparison with participants with a Dutch background in The Netherlands was explained by differences in two markers of socioeconomic status (education and neighbourhood) [27]. Considering the small number of CT-positive participants in our sample, it was not possible examine the associations of sociodemographic characteristics and sexual behaviour and the occurrence of CT infection.

The strengths of our study are that we were successful in recruiting a considerable number of respondents from the hard-to-reach Roma population living in settlements. This was achieved by following the principles of community-based participatory research through engaging Roma community workers. Our study also has some limitations. Data on behaviour were collected differently among Roma and non-Roma, by interview and by questionnaire, respectively. Our sample was representative for Roma living in separated and segregated settlements. Therefore, the results can be generalised only to this most vulnerable part of Roma population, whose sexual risk behaviour might differ from the behaviour of Roma who are integrated among the majority population in towns or villages. Another limitation of this study is the focus only on urogenital chlamydial infection. We did not collect samples from oral/pharyngeal or rectal anatomical sites, potentially resulting in an underestimate of chlamydia prevalence. Future research should be extended to explore sexual practices (vaginal, anal, oral sex, provision of sexual commercial services, men who have sex with men) and extragenital screening including the oropharynx and rectum. Future studies might cover wider spectrum of substance use which might be associated with risky sexual behaviour and consequently with sexually transmitted infections. Sexual behaviour may be affected not only by the use of alcohol or other substances but also by socio-economic status or psychosocial determinants, which should be explored further. Nevertheless, as pointed out in the study of Madarasova Geckova et al. (2014) “the deteriorating effect of living in Roma settlement on health and health related behaviour seems to be immense regardless differences in SE characteristics or living conditions within the settlement population” [4].

## 5. Conclusions

Although Roma do not differ in the prevalence of CT infection compared to the majority population, they display more risky behaviour in terms of earlier initiation of sex life and less prevalent consistent condom use. Moreover, this population face barriers to health care which may increase the opportunity to spread infection in case of delayed diagnosis and treatment. Therefore, measures focusing on screening and enhancing (empowering) in relevant health care should be applied [9,67].

## Figures and Tables

**Table 1:** Basic characteristics of the Roma and non-Roma samples and *p*-values for differences between the Roma and non-Roma population in eastern Slovakia.

	Roma	Non-Roma	*p*-Value
	*n* = 452	*n* = 403	
Age, mean (SD)	34.7 (9.14)	33.5 (7.4)	<0.05 ^a^
Gender, *n* (%)			
Women	293 (64.8)	218 (54.1)	<0.01 ^b^
Men	159 (35.2)	185 (45.9)	
Highest education, *n* (%)			
Higher	10 (2.3)	300 (76.3)	<0.001 ^b^
Apprenticeship	73 (16.5)	84 (21.4)	
Elementary	360 (81.3)	9 (2.3)	
Unemployment, *n* (%)	396 (89.6)	102 (26.4)	<0.001 ^b^
Number of children in household, mean (SD)	3.8 (3.72)	1.2 (1.48)	<0.001 ^a^

^a^ *t*-test, ^b^ Chi-square test. SD: standard deviation.

**Table 2:** Sexual and drinking behaviour of the population living in Roma settlements and the non-Roma population in eastern Slovakia (logistic regression model adjusted for age, odds ratios and 95% confidence intervals).

	Roma *n* (%)	Non-Roma ^1^ *n* (%)	OR (CI)
Living with a partner in the present			
Yes	374 (82.7)	263 (65.3)	2.48 (1.77–3.47) ***
Age at first sexual intercourse			
15 years or less	126 (27.9)	18 (4.5)	7.71 (4.59–12.94) ***
Number of sexual partners			
5 or more partners	52 (11.5)	83 (20.6)	0.45 (0.31–0.66) ***
Condom use			
Inconsistent use	422 (93.4)	314 (77.9)	3.56 (1.99–6.36) ***
Reasons for condom use			
Protection against unwanted pregnancy	103 (22.8)	177 (43.9)	0.37 (0.28–0.50) ***
Protection against infectious diseases	40 (8.8)	88 (21.8)	0.34 (0.23–0.52) ***
Sexual intercourse for money or reward			
Yes	13 (2.9)	8 (2.0)	1.37 (0.56–3.34) ^n.s.^
Drinking behaviour			
6 or more doses of alcohol on one occasion once a month or more	75 (16.6)	64 (15.6)	1.06 (0.74–1.54) ^n.s.^

*** *p* < 0.001, n.s. nonsignificant difference; ^1^ the reference group for logistic regression. OR: odds ratio; CI: confidence interval.

**Table 3:** Sexual and drinking behaviour of the population living in Roma settlements and the non-Roma population in eastern Slovakia (logistic regression model, odds ratios and 95% confidence intervals).

	Drinking Behaviour (Six or More Doses of Alcohol on One Occasion)		
	Less Than Once a Month ^1^ *n* (%)	Once a Month or More *n* (%)	OR (CI)
ROMA			
Number of sexual partners			
Five or more partners	26 (7.1)	26 (34.7)	6.99 (3.73–13.09) ***
Condom use			
Inconsistent use	347 (94.8)	69 (92)	0.67 (0.21–2.14) ^n.s.^
NON-ROMA			
Number of sexual partners			
Five or more partners	54 (16.7)	29 (45.3)	4.27 (2.35–7.78) ***
Condom use			
Inconsistent use	256 (79)	55 (85.9)	1.77 (0.70–4.49) ^n.s.^

*** *p* < 0.001, ^n.s.^ non-significant difference; ^1^ the reference group for logistic regression.

**Table 4:** Occurrence of *Chlamydia trachomatis* infection in the population living in Roma settlements and the non-Roma population in eastern Slovakia.

	Roma *N* = 422	Non-Roma *N* = 335	*p*-Value ^a^
Men, *n* (%)	3 (2.0)	4 (2.4)	0.778
Women, *n* (%)	13 (4.8)	5 (2.9)	0.330
∑	16 (3.8)	9 (2.7)	0.398

^a^ Chi-square test.
